# Shared Decision‐Making and Trajectories of Self‐Management Confidence in Nurse‐Led Chronic Condition Care: A Longitudinal Evaluation

**DOI:** 10.1111/jan.70634

**Published:** 2026-05-07

**Authors:** Jude Ominyi, Aaron Nwedu, David Agom, Anastasia Ngon

**Affiliations:** ^1^ School of Health, Sciences & Society University of Suffolk Ipswich UK; ^2^ Department of Nursing Science David Umahi Federal University of Health Sciences Uburu Ebonyi State Nigeria; ^3^ Faculty of Health, Sports and Behavioural Sciences University of Northampton Northampton UK; ^4^ Faculty of Business & Law University of Northampton Northampton UK

## Abstract

**Aim:**

To examine whether shared decision‐making at baseline is associated with trajectories of self‐management confidence over 12 months among individuals living with chronic conditions attending nurse‐led clinics.

**Design:**

A longitudinal design.

**Methods:**

Individuals with at least one clinician confirmed chronic condition were recruited from six nurse‐led primary care clinics between March and September 2022. Data collection took place between March 2022 and September 2023, with baseline, 6 and 12‐month assessments completed within routine follow‐up contacts. Self‐management confidence was measured using the Self‐Efficacy for Managing Chronic Disease Scale, and perceived shared decision‐making was assessed using the nine‐item Shared Decision‐Making Questionnaire (SDM‐Q‐9). Linear mixed‐effects modelling examined changes in confidence over time and associations with baseline shared decision‐making, adjusting for age, gender, education and number of chronic conditions.

**Results:**

Of 157 eligible individuals approached, 151 consented to participate (96.2%), and 146 were retained at 12 months (96.7%). Mean self‐management confidence increased from 40.2 at baseline to 44.5 at 12 months. In adjusted models, confidence was significantly higher at 6 months (*β* = 2.63, 95% CI: 1.54–3.72, *p* < 0.001) and 12 months (*β* = 4.21, 95% CI: 2.93–5.49, *p* < 0.001) compared with baseline. Higher baseline shared decision‐making was positively associated with repeated confidence scores across follow‐up (*β* = 0.10, 95% CI: 0.04–0.16, *p* = 0.002). The association was stronger among participants aged under 60 years.

**Conclusions:**

Within established nurse‐led chronic condition care, perceived shared decision‐making was statistically associated with subsequent trajectories of self‐management confidence over 12 months. Confidence demonstrated gradual change rather than stability within routine practice.

**Implications:**

Strengthening the visibility and consistency of collaborative dialogue within nurse‐led consultations may support self‐management confidence. Structured conversational approaches that help nurses enact shared decision‐making more explicitly warrant further evaluation in primary care.

**Patient or Public Contribution:**

No patients were involved in study design.

## Summary

- What does this paper contribute to the wider global clinical nursing community?
  ○ Provides longitudinal evidence from routine nurse‐led care showing that perceived shared decision‐making is associated with trajectories of self‐management confidence over 12 months.
  ○ Extends understanding of relational processes in chronic condition management beyond predominantly cross‐sectional evidence, highlighting confidence as dynamic within ongoing care relationships.
  ○ Supports closer attention to how collaborative dialogue is structured and enacted in nurse‐led consultations as a potentially important dimension of chronic condition care.

## 1 Introduction

Chronic conditions account for a substantial and growing proportion of global morbidity, healthcare utilisation and expenditure (World Health Organisation [WHO] 2024). Individuals living with one or more chronic conditions frequently manage persistent symptoms, complex treatment regimens and care decisions that extend across clinical settings (Coulter et al. 2015). While multimorbidity, defined as two or more co‐existing chronic illnesses, is increasingly recognised within policy and research (Ominyi et al. 2025), individuals living with a single chronic condition also encounter uncertainty and must interpret bodily changes and decide when professional input is required (British Heart Foundation 2023). Confidence in managing illness has therefore become central to contemporary chronic care frameworks, given its association with adherence, symptom control and service engagement (Iroegbu et al. 2025). In the United Kingdom, supported self‐management and personalised care are core components of primary care delivery for chronic conditions (National Institute for Health and Care Excellence (NICE) 2018, 2025). Nurse‐led clinics are central to this model. Despite strong policy emphasis on collaborative practice within UK primary care, longitudinal evidence examining how perceived shared decision‐making within routine nurse‐led chronic condition services relates to self‐management confidence over time remains rare. This study addresses that gap by examining statistical associations between baseline shared decision‐making and trajectories of self‐management confidence across 12 months in established nurse‐led primary care.

## 2 Background

Self‐management confidence is commonly conceptualised through self‐efficacy theory, which defines self‐efficacy as an individual's belief in their capability to organise and execute actions required to manage specific challenges (Bandura 1997). Within chronic conditions, higher self‐efficacy has been associated with engagement in self‐management behaviours, adaptive coping and perceived control over illness trajectories (Iroegbu et al. 2025). Confidence is not solely an internal attribute (Dinh and Bonner 2023; Badr et al. 2024), it may also be shaped through interpersonal experiences within healthcare encounters. Communication that is responsive, respectful and tailored has been associated with patient‐reported outcomes in chronic condition contexts (Iroegbu et al. 2025).

Shared decision‐making represents one structured form of collaborative communication (Dinh and Bonner 2023), and involves clinicians and patients working together to consider options, deliberate on preferences and agree on care plans aligned with patients' values (Perron et al. 2024). Evidence suggests that shared decision‐making is associated with improved perception of deliberation quality and involvement in care planning (Kriston et al. 2010; Perron et al. 2024). Observational and intervention studies have also explored associations with self‐management‐related outcomes, although findings vary across populations and measures (Chang et al. 2023; McDonagh et al. 2021). Patient characteristics such as self‐efficacy and communication experiences appear relevant to engagement in decision processes (Keij et al. 2023).

Nurse‐led clinics are a key setting for structured follow‐up of individuals living with chronic conditions, including diabetes, chronic obstructive pulmonary disease, heart failure and hypertension (Liddy et al. 2024). Continuity of contact may allow iterative clarification of concerns, adjustment of plans and personalised dialogue across repeated consultations (Friesen‐Storms et al. 2014; Ominyi et al. 2025). National guidance emphasises supported self‐management, collaborative care planning and regular review as central to high‐quality chronic condition management (NICE 2018, 2025; NHS England 2025). For example, heart failure guidance highlights planned follow‐up and patient engagement in symptom interpretation to reduce avoidable deterioration (British Heart Foundation 2023). Despite this policy and practice context, most research examining shared decision‐making and confidence remains cross sectional or short term (Chang et al. 2023; Perron et al. 2024). A prospective longitudinal design could facilitate the examination of whether baseline perceptions of shared decision‐making are statistically associated with subsequent patterns of self‐management confidence within established care relationships. Such an approach may contribute empirical insight into relational processes within chronic condition care while maintaining conceptual clarity and avoiding causal claims.

## 3 The Study

### 3.1 Aim

This study aimed to examine the association between perceived shared decision‐making and trajectories of self‐management confidence over 12 months among individuals living with chronic conditions attending nurse‐led clinics.

### 3.2 Research Questions

1. Is baseline perceived shared decision‐making statistically associated with repeated measures of self‐management confidence over 12 months among individuals living with chronic conditions?
2. Does self‐management confidence change over time within established nurse‐led chronic condition care?
3. Do associations between baseline shared decision‐making and self‐management confidence trajectories vary according to patient characteristics, including age, education level, number of chronic conditions or baseline confidence?

## 4 Method

### 4.1 Design

A longitudinal design was utilised in this study. Longitudinal designs are appropriate where the aim is to examine temporal associations within real‐world practice without manipulating exposure or care delivery (Flanagan et al. 2025; Twisk et al. 2018; von Elm et al. 2007). Repeated measurement of self‐management confidence at baseline, 6 months and 12 months enabled assessment of within‐person change over time while preserving the natural context of nurse‐led care. Baseline represented the start of study measurement rather than initiation of the nurse–patient relationship, as participants had already been engaged in nurse‐led care prior to enrolment. This distinction is important in interpreting trajectories, as early relational development was not captured within the study window. The study was reported in accordance with the Strengthening the Reporting of Observational Studies in Epidemiology (STROBE) guidance for cohort studies (von Elm et al. 2007).

### 4.2 Sample and Settings

The study was conducted across six nurse‐led primary care clinics providing structured follow‐up for individuals living with chronic conditions. Clinics delivered routine review appointments, medication monitoring, symptom assessment and ongoing consultation in line with national policy expectations for chronic condition management. Nurse‐led models are a prominent feature of chronic condition care, particularly in diabetes, chronic obstructive pulmonary disease, heart failure and hypertension services, where continuity and relational dialogue are central components of care delivery. Examining associations within this context therefore reflects routine practice rather than an enhanced or experimental service configuration.

Eligible participants were adults aged 18 years or older with a clinician‐confirmed diagnosis of at least one chronic condition and active engagement with the nurse‐led clinic for a minimum of 3 months prior to enrolment. This criterion was applied to ensure that baseline measures reflected perceptions formed within an established therapeutic relationship rather than first‐contact impressions. Individuals receiving end‐of‐life care or lacking capacity to provide informed consent were excluded.

Recruitment occurred between March and September 2022 and was embedded within scheduled clinic appointments. Clinic staff identified potentially eligible patients from appointment lists prior to routine review visits. Eligible patients were then approached during their scheduled appointment, provided with study information and invited to participate by a member of the research team. Those who agreed completed consent procedures and baseline questionnaires during the same episode of care. Embedding recruitment within routine appointments reduced additional participant burden and aligned study procedures closely with usual service delivery. The overall study period, including 6‐ and 12‐month follow‐up, extended from March 2022 to September 2023. Of 157 eligible patients approached, 151 consented to participate (96.2%). Follow‐up data were available for 151 participants at 6 months and 146 at 12 months. Further details regarding participant characteristics are presented in Section 5.1.

### 4.3 Data Collection

Data were collected at three time points: baseline (study enrolment), 6 months and 12 months. Baseline data were collected during recruitment between March and September 2022, and follow‐up data collection continued until September 2023. Baseline questionnaires were completed during routine clinic visits. Follow‐up questionnaires were aligned, wherever possible, with scheduled review appointments in order to minimise disruption and support continuity of participation. Where participants were unable to complete follow‐up during an in‐person clinic contact, questionnaires were completed through agreed alternative arrangements consistent with local study procedures. Participants had been engaged in nurse‐led care for at least 3 months prior to enrolment; therefore, baseline represents the start of study measurement rather than the beginning of the clinical relationship. Follow‐up was integrated within existing care pathways, with contact maintained through routine appointment systems in order to minimise additional participant burden and support continuity of participation. Nurses involved in direct clinical care were not involved in data entry or statistical analysis in order to reduce the risk of role‐related bias.

### 4.4 Measures

Validated self‐report instruments were used to assess study variables. Selection of measures was informed by their prior application in chronic illness research and evidence of acceptable reliability and validity in adult populations living with chronic conditions. For example, the Self‐Efficacy for Managing Chronic Disease Scale has demonstrated good internal consistency and construct validity in chronic disease populations (Lorig et al. 2001), while the SDM‐Q‐9 has shown good psychometric performance in assessing patients' perceptions of involvement in decision‐making across a range of clinical settings.

#### 4.4.1 Demographic and Clinical Characteristics

Participants reported age, gender, ethnicity, highest educational qualification, employment status and living arrangement. Clinical characteristics included primary diagnosis, number of chronic conditions and duration of enrolment within the nurse‐led clinic. Multimorbidity was defined as the presence of two or more chronic conditions.

#### 4.4.2 Self‐Management Confidence

Self‐management confidence was assessed using the Self‐Efficacy for Managing Chronic Disease Scale (Lorig et al. 2001). This six‐item instrument asks respondents to rate their confidence in managing symptoms, fatigue, emotional distress and other illness‐related challenges on a 10‐point scale ranging from 1 (not at all confident) to 10 (totally confident). Total scores range from 6 to 60, with higher scores indicating greater self‐management confidence. The scale has demonstrated good internal consistency and construct validity in chronic illness populations (Lorig et al. 2001) and is conceptually grounded in self‐efficacy theory (Bandura 1997). In the present sample, internal consistency was assessed using Cronbach's alpha.

#### 4.4.3 Shared Decision‐Making

Perceived shared decision‐making was measured using the nine‐item Shared Decision‐Making Questionnaire (SDM‐Q‐9) (Härter et al. 2021). The SDM‐Q‐9 evaluates the extent to which patients perceive involvement in clinical decision processes during consultations. Items are rated on a 6‐point Likert scale and transformed to a 0–100 scale, with higher scores reflecting greater perceived shared decision‐making. The SDM‐Q‐9 has demonstrated good reliability and validity across chronic care settings (Härter et al. 2021). Internal consistency in the present sample was evaluated using Cronbach's alpha.

### 4.5 Data Analysis

Descriptive statistics summarised demographic and clinical characteristics at baseline. Continuous variables are presented as means with standard deviations, and categorical variables as frequencies and percentages. Internal consistency of the Self‐Efficacy for Managing Chronic Disease Scale and the SDM‐Q‐9 was assessed in the present sample using Cronbach's alpha coefficients. Longitudinal associations were examined using linear mixed‐effects modelling. Mixed‐effects models are appropriate for repeated measures data because they account for within‐person correlation over time and allow inclusion of participants with incomplete follow‐up under maximum likelihood estimation (Twisk et al. 2018). All available observations were included in the models.

Time was modelled as a categorical variable with baseline as the reference category. Three sequential models were specified to address the research questions. Model 1 examined change in self‐management confidence over time. Time indicators (6 and 12 months) were entered to estimate differences in confidence relative to baseline. Model 2 added baseline shared decision‐making as a predictor variable to examine its association with repeated measures of self‐management confidence. Age, gender, education level and number of chronic conditions were included as covariates. Ethnicity and duration of engagement with the clinic were examined descriptively but were not included in the adjusted models. Ethnicity was not retained because several subgroup counts were small, which risked unstable parameter estimates and reduced interpretability. Duration of clinic engagement was also not retained in the final models because baseline was explicitly conceptualised as the start of study measurement within an already established care relationship rather than as the start of exposure to the service. Model 3 included interaction terms between baseline shared decision‐making and selected participant characteristics (age group, education level, multimorbidity status and baseline confidence) to examine whether associations differed across subgroups. Interaction terms were retained only if statistically significant. Estimates are presented as unstandardised regression coefficients (*β*) with 95% confidence intervals. Statistical significance was set at *p* < 0.05 (two‐sided). Model assumptions were examined through inspection of residual distributions and plots of fitted values.

### 4.6 Ethical Considerations

Ethical approval was given by the University Research Ethics Committee (Reference #00145, approval date 02/02/2022). In addition, the project was reviewed through local governance processes within participating clinics and was managed locally as service evaluation. The project did not involve randomisation, alteration to treatment or the introduction of additional clinical procedures, and data were collected through participant‐completed questionnaires embedded within routine nurse‐led follow‐up care. In line with UK Health Research Authority guidance, projects that are not managed as research do not require HRA Approval or NHS Research Ethics Committee review; instead, local governance arrangements apply. All participants received written information, provided informed consent and were assured that participation would not affect their care.

## 5 Results

### 5.1 Feasibility and Participants' Demographic Characteristics

Of 157 eligible individuals approached, 151 consented to participate (96.2%). Follow‐up completion was 100% at 6 months and 96.7% at 12 months (*n* = 146). Item‐level missing data were minimal (< 2% per scale at baseline and follow‐up). Linear mixed‐effects models included all available observations under maximum likelihood estimation. Baseline reflected the start of study measurement rather than initiation of the nurse–patient relationship, as participants had been engaged in nurse‐led care for at least 3 months prior to enrolment.

Participants had a mean age of 62.4 years (SD = 12.1), with a range of 29 to 87 years. Slightly more than half were female (56.9%). Just over half, 51.7%, were living with multimorbidity, defined as two or more co‐existing chronic conditions. Participants had been enrolled in the nurse‐led clinic for a mean of 18.6 months (SD = 11.2), indicating that baseline measures reflected experiences formed within established service relationships.

Baseline self‐management confidence averaged 40.2 (SD = 9.1). Participants with multimorbidity reported lower confidence than those with a single chronic condition (mean difference = 3.6, *p* = 0.004). Mean shared decision‐making score at enrolment was 69.1 (SD = 14.2). Participant demographic and clinical characteristics are summarised in Table 1. Although ethnicity data were collected and are reported descriptively to characterise the sample, ethnicity was not included in adjusted models because several subgroup counts were small, limiting statistical stability for multivariable estimation.

**TABLE 1:** Participant characteristics at baseline (*n* = 151).

Variable	Value
Age, mean ± SD	62.4 ± 12.1
Gender	
Female	86 (56.9%)
Male	65 (43.1%)
Ethnicity	
White British	110 (72.8%)
South Asian	20 (13.2%)
Black African/Caribbean	10 (6.6%)
Other or mixed	11 (7.4%)
Highest educational qualification	
No formal qualification	16 (10.6%)
Secondary education	63 (41.7%)
College or university degree	72 (47.7%)
Employment status	
Employed	57 (37.7%)
Retired	58 (38.4%)
Unemployed	24 (15.9%)
Other	12 (8.0%)
Living arrangement	
Living alone	44 (29.1%)
Living with others	107 (70.9%)
Primary diagnosis	
Type 2 diabetes	69 (45.7%)
Chronic obstructive pulmonary disease	41 (27.2%)
Heart failure	26 (17.2%)
Other chronic conditions	15 (9.9%)
Number of conditions	
Multimorbidity (≥ 2 conditions)	78 (51.7%)
Single condition	73 (48.3%)
Duration of enrolment within nurse‐led clinic, mean ± SD (months)	18.6 ± 11.2
Baseline self‐management confidence, mean ± SD	40.2 ± 9.1
Shared decision‐making score, mean ± SD	69.1 ± 14.2

*Note:* Values are mean ± standard deviation (SD) or *n* (%). Multimorbidity was defined as two or more chronic conditions.

### 5.2 Change in Self‐Management Confidence Over Time (RQ2)

Model 1 examined change in self‐management confidence across the three time points. Observed mean scores increased from 40.2 (SD = 9.1) at baseline to 42.8 (SD = 8.7) at 6 months and 44.5 (SD = 8.3) at 12 months. In the mixed‐effects model adjusted for age, gender, education and number of chronic conditions, time was significantly associated with self‐management confidence. Compared with baseline, confidence was higher at 6 months (*β* = 2.63, 95% CI: 1.54–3.72, *p* < 0.001) and 12 months (*β* = 4.21, 95% CI: 2.93–5.49, *p* < 0.001). Adjusted estimated means with 95% confidence intervals are presented in Table 2 and illustrated in Figure 1.

**TABLE 2:** Adjusted estimated means for self‐management confidence.

Time	Mean	SE	95% CI
Baseline	40.2	0.74	38.7–41.7
6 months	42.8	0.71	41.4–44.2
12 months	44.5	0.69	43.1–45.9

**FIGURE 1:**
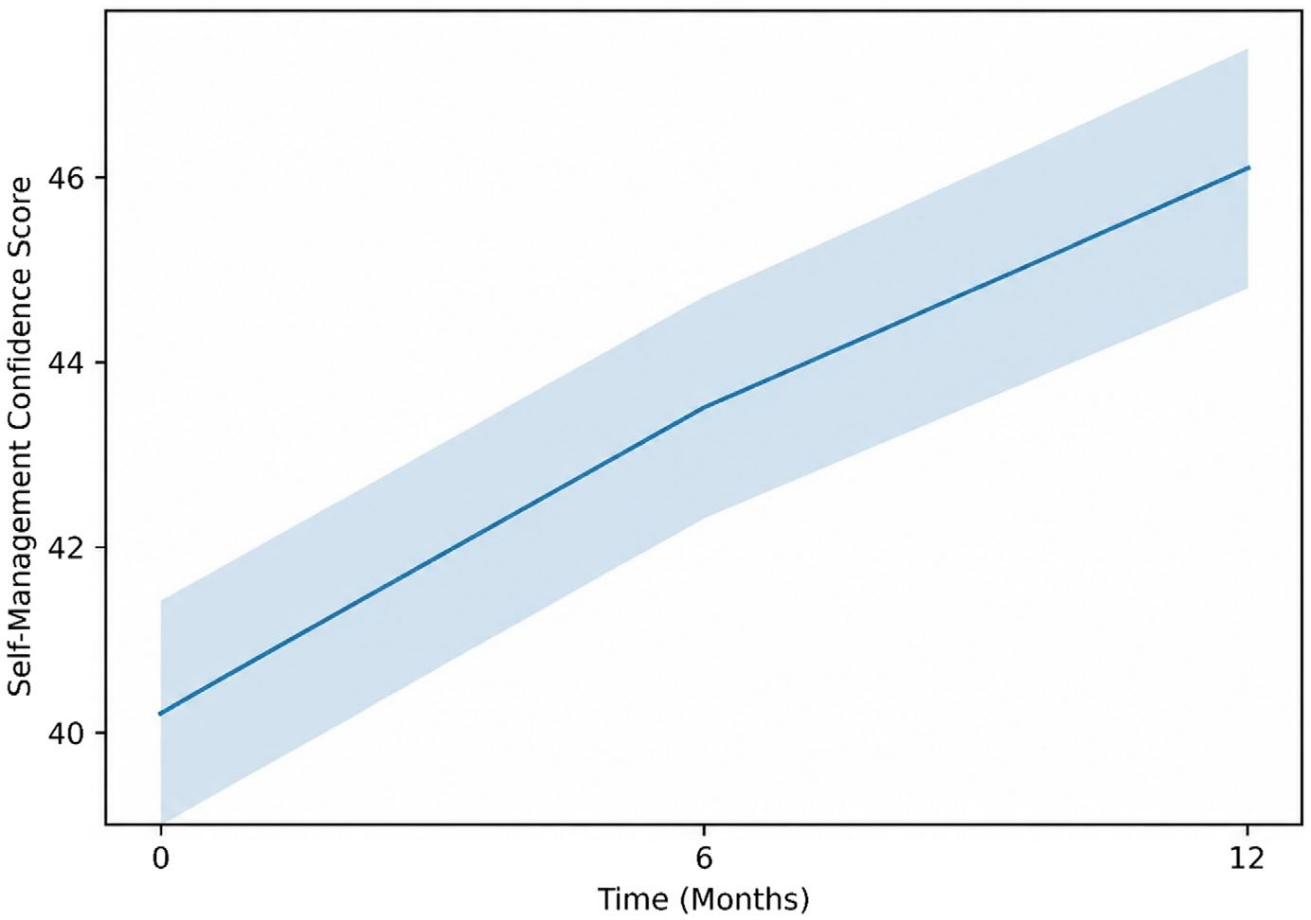
Adjusted trajectory of self‐management confidence.

### 5.3 Association Between Baseline Shared Decision‐Making and Confidence Trajectories (RQ1)

Model 2 added baseline shared decision‐making to the time model. After adjustment for age, gender, education level and number of chronic conditions, baseline shared decision‐making was positively associated with repeated self‐management confidence scores (*β* = 0.10, 95% CI: 0.04–0.16, *p* = 0.002). Each one‐point higher baseline shared decision‐making score was associated with a 0.10‐point higher self‐management confidence score across follow‐up. The association remained stable after inclusion of covariates. Full model estimates are presented in Table 3.

**TABLE 3:** Mixed‐effects model of self‐management confidence including shared decision‐making.

Predictor	*β*	95% CI	*p*
Shared decision‐making	0.10	0.04–0.16	0.002
6 months (vs. baseline)	2.63	1.54–3.72	< 0.001
12 months (vs. baseline)	4.21	2.93–5.49	< 0.001
Multimorbidity	−2.74	−4.71 to −0.77	0.007

Predicted trajectories at the 25th and 75th percentiles of baseline shared decision‐making are displayed in Figure 2.

**FIGURE 2:**
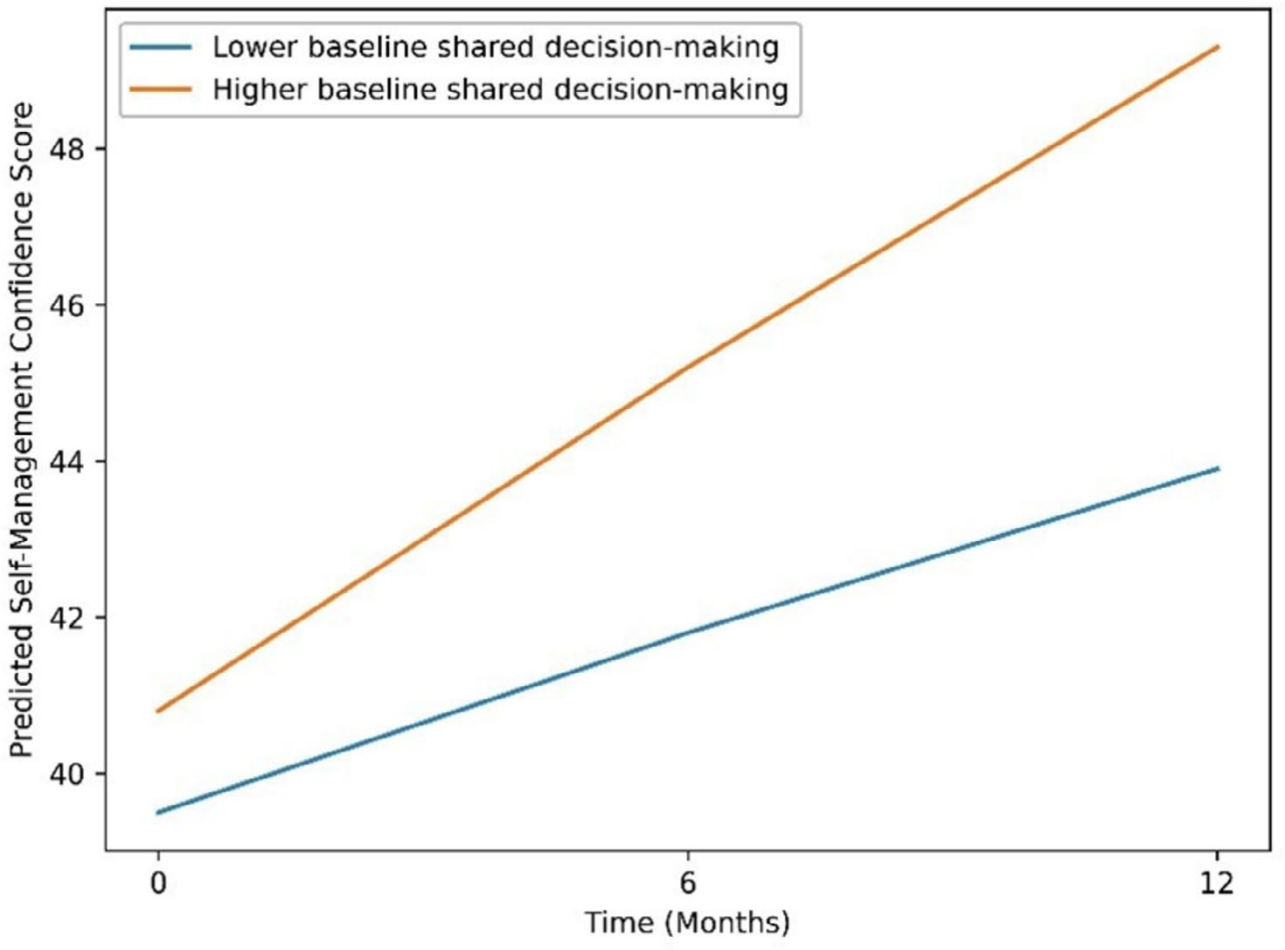
Predicted trajectories by baseline shared decision‐making.

### 5.4 Variation in Associations by Patient Characteristics (RQ3)

Model 3 examined interaction terms between shared decision‐making and selected participant characteristics. A statistically significant interaction was observed between shared decision‐making and age group (< 60 vs. ≥ 60 years) (*β* = 0.12, 95% CI: 0.02–0.22, *p* = 0.018), indicating that the association between shared decision‐making and self‐management confidence was stronger among participants aged under 60 years. Interactions between shared decision‐making and multimorbidity (*p* = 0.173), education (*p* = 0.214) and baseline confidence (*p* = 0.241) were not statistically significant. Interaction coefficients are presented in Table 4.

**TABLE 4:** Interaction effects between baseline shared decision‐making and selected participant characteristics on repeated self‐management confidence scores.

Interaction term	*β*	95% CI	*p*
SDM × Age (< 60 vs. ≥ 60 years)	0.12	0.02 to 0.22	0.018
SDM × Multimorbidity	0.07	−0.03 to 0.17	0.173
SDM × Education	0.05	−0.03 to 0.13	0.214
SDM × Baseline confidence	0.03	−0.02 to 0.08	0.241

*Note:* Positive coefficients indicate a stronger association between baseline shared decision‐making and repeated self‐management confidence scores in the relevant subgroup.

Abbreviations: *β*, unstandardised regression coefficient; CI, confidence interval; SDM, shared decision‐making.

In summary, self‐management confidence scores were higher at 6 and 12 months than at baseline within this cohort attending nurse‐led chronic care. The association between shared decision‐making and repeated confidence scores was stronger among younger participants and did not vary significantly according to education level, multimorbidity status or baseline confidence.

## 6 Discussion

This longitudinal cohort study examined whether perceived shared decision‐making at study enrolment was statistically associated with trajectories of self‐management confidence over 12 months among individuals living with chronic conditions. Three key findings emerged. First, self‐management confidence scores were higher at 6 and 12 months compared with baseline. Second, higher baseline shared decision‐making was positively associated with repeated confidence scores across follow‐up. Third, the strength of this association varied by age but did not differ significantly according to education level, multimorbidity status or baseline confidence.

Self‐management confidence increased over the 12‐month period within this cohort receiving structured nurse‐led care. Observed improvements were modest in magnitude but consistent across time points. These findings align with longitudinal studies indicating that confidence in managing chronic illness can evolve during ongoing engagement with primary care services (Dinh and Bonner 2023; Iroegbu et al. 2025). Previous studies have reported that repeated contact, review appointments and opportunities for discussion may be associated with gradual strengthening of patients' perceived capability to manage symptoms and treatment regimens (Ominyi et al. 2025). Findings should not be interpreted as evidence that nurse‐led care directly increased confidence, as baseline represented the start of measurement rather than the beginning of the therapeutic relationship. Participants had already been engaged in care for at least 3 months, and on average substantially longer. Early development of confidence may therefore have occurred prior to study enrolment and remains outside the scope of observation. Results instead describe confidence trajectories within an established care context.

Participants living with multimorbidity reported lower baseline confidence compared with those with a single chronic condition. This pattern is consistent with evidence that treatment burden and symptom complexity are greater among individuals with multiple conditions (Boehmer et al. 2018; WHO 2024). Lower baseline confidence in this group likely reflects the practical and interpretive demands of managing overlapping symptoms, multiple treatments and competing self‐management priorities rather than differential exposure to care within the study.

Higher perceived shared decision‐making at baseline was statistically associated with higher repeated confidence scores over time. The magnitude of the association was modest but remained consistent after adjustment for demographic and clinical covariates. This finding aligns with work linking collaborative communication with patient‐reported outcomes in chronic illness populations (Härter 2020; Iroegbu et al. 2025; Perron et al. 2024; Eynan et al. 2024). Findings of this study extend that literature by showing that perceived involvement in decision‐making within routine nurse‐led care is associated with confidence trajectories across a 12‐month period rather than only at a single time point. However, interpretation should remain cautious; behavioural outcomes were not directly measured, and confidence represents a self‐reported perception grounded in self‐efficacy theory (Bandura 1997). The observed association does not establish that shared decision‐making produces changes in behaviour or clinical outcomes. Rather, findings suggest that individuals who perceive greater collaboration at enrolment also report higher confidence scores during follow‐up. Longitudinal evidence in this area remains limited. Much of the existing literature is cross‐sectional (Chang et al. 2023) or short term (Perron et al. 2024).

The association between shared decision‐making and confidence was statistically stronger among participants under 60 years of age. Younger adults may place greater emphasis on participatory communication styles or may differ in expectations regarding involvement in care decisions (Keij et al. 2023). Age‐related variation in preferences for involvement has been reported previously, although findings remain mixed and context dependent (Perron et al. 2024). No statistically significant variation was observed according to education level, multimorbidity status or baseline confidence. These findings suggest that the association between perceived collaboration and confidence is not confined to individuals with higher educational attainment or those with a particular starting level of confidence. Such consistency is relevant for nurse‐led services serving socially and clinically diverse populations. Absence of significant interaction with multimorbidity indicates that relational communication may be similarly associated with confidence among individuals with single or multiple conditions. Given the greater treatment complexity experienced by people with multimorbidity (Boehmer et al. 2018), this finding warrants further investigation in larger samples.

This study contributes to the literature in three ways. First, it provides longitudinal evidence from routine nurse‐led clinics rather than an intervention or experimental context. Second, it demonstrates that perceived shared decision‐making is associated with confidence trajectories over a clinically meaningful period. Third, it shows that this association is broadly consistent across educational and multimorbidity groups, with some age‐related variation. Existing studies have often focused on decisional conflict, satisfaction or knowledge outcomes (Perron et al. 2024; Gagliardi et al. 2016). Fewer studies have examined how collaborative communication relates to longer‐term perceptions of capability within chronic condition care. Findings therefore extend current understanding of relational processes in nurse‐led chronic condition services.

### 6.1 Limitations

A few limitations merit consideration. Baseline measurement occurred after participants had already established relationships with nurses. Early development of confidence and relational perceptions was therefore not captured. Self‐report instruments may be subject to recall or social desirability bias. Effect sizes were modest, and clinical significance should be interpreted cautiously. Residual confounding cannot be excluded, as unmeasured factors such as illness severity, prior education for self‐management or broader social support were not included. Ethnicity was collected but not entered into adjusted models because several subgroup counts were small, limiting stable estimation; consequently, potential ethnic differences in experiences of shared decision‐making and confidence could not be examined. Despite these limitations, retention was high, missing data were minimal and mixed‐effects modelling allowed inclusion of all available observations. These methodological strengths enhance confidence in the stability of the reported associations.

## 7 Implications for Practice and Research

Findings have implications for nurse‐led chronic condition services. Individuals who perceived greater collaboration reported higher confidence across follow‐up. While causal inference cannot be made, the results suggest that the quality and structure of consultation dialogue warrant continued attention within routine practice. Structured conversational approaches may assist nurses in making shared decision‐making more explicit and reproducible within time‐constrained consultations. One possible example is a TGROW‐informed coaching. TGROW is an adaptation of the GROW coaching framework, commonly described as Goal, Reality, Options and Will, with the additional ‘T’ used to establish the topic or focus of the conversation at the outset (Whitmore 2019; Wilson 2020). Within chronic condition reviews, such frameworks may help patients articulate priorities, consider options and connect clinical advice to day‐to‐day self‐management. However, this study does not demonstrate the effectiveness of TGROW specifically, nor does it justify privileging one structured approach over others. The findings instead support broader empirical attention to coaching‐informed or otherwise structured models that may strengthen collaborative communication in nurse‐led chronic condition care.

Future studies should therefore examine the feasibility, acceptability and potential effects of structured conversational approaches within UK nurse‐led chronic condition clinics. Comparative work would be particularly valuable, rather than focusing prematurely on a single model. Prospective studies could assess whether changes in perceived shared decision‐making over time correspond with changes in confidence. Cluster randomised feasibility trials, stepped‐wedge designs or mixed methods process evaluations may be appropriate approaches for examining implementation and mechanism. Future studies should also incorporate behavioural and clinical outcomes alongside confidence measures in order to determine whether relational processes are associated with observable changes in self‐management practices, healthcare utilisation or symptom control.

## 8 Conclusion

Chronic condition management within the UK increasingly emphasises relational continuity and supported self‐management. Longitudinal evidence examining how perceived collaboration relates to evolving confidence within routine nurse‐led services has remained limited. Findings from this study indicate that perceptions of shared decision‐making are statistically associated with subsequent confidence trajectories over 12 months in established care relationships. Confidence demonstrated gradual change rather than stability, reinforcing the view that perceived capability in chronic condition management is dynamic. These findings support continued empirical attention to consultation quality within nurse‐led primary care. Strengthening the structure and visibility of collaborative dialogue may assist alignment between policy ambitions and everyday clinical practice. Future research integrating relational measures with behavioural and clinical outcomes will be important in advancing nursing understanding of how consultation processes relate to adaptation and self‐management in chronic condition care.

## Author Contributions

Jude Ominyi, Aaron Nwedu and David Agom: made substantial contributions to conception and design, or acquisition of data, or analysis and interpretation of data; Jude Ominyi, Aaron Nwedu, David Agom, Anastasia Ngon: involved in drafting the manuscript or revising it critically for important intellectual content; given final approval of the version to be published. Each author should have participated sufficiently in the work to take public responsibility for appropriate portions of the content; agreed to be accountable for all aspects of the work in ensuring that questions related to the accuracy or integrity of any part of the work are appropriately investigated and resolved.

## Funding

The authors have nothing to report.

## Disclosure

Confirm that any data utilised in the submitted manuscript have been lawfully acquired in accordance with The Nagoya Protocol on Access to Genetic Resources and the Fair and Equitable Sharing of Benefits Arising from Their Utilisation to the Convention on Biological Diversity. State that the relevant fieldwork permission was obtained and list the permit numbers.

## Ethics Statement

Ethical approval was given by the University Research Ethics Committee (Reference #00145, approval date 02/02/2022). In addition, the project was reviewed through local governance processes within participating clinics and was managed locally as service evaluation. The project did not involve randomisation, alteration to treatment or the introduction of additional clinical procedures, and data were collected through participant‐completed questionnaires embedded within routine nurse‐led follow‐up care. In line with UK Health Research Authority guidance, projects that are not managed as research do not require HRA Approval or NHS Research Ethics Committee review; instead, local governance arrangements apply. All participants received written information, provided informed consent and were assured that participation would not affect their care.

## Consent

Informed consent to participate was obtained from all participants prior to their enrolment in this study. Participant information sheets and consent forms were provided in advance.

## Conflicts of Interest

We declare no conflicts of interest.

## Data Availability

All data generated from this study have been presented in the manuscript.
